# Dendritic Cell-Specific Delivery of Flt3L by Coronavirus Vectors Secures Induction of Therapeutic Antitumor Immunity

**DOI:** 10.1371/journal.pone.0081442

**Published:** 2013-11-28

**Authors:** Christian Perez-Shibayama, Cristina Gil-Cruz, Monika Nussbacher, Eva Allgäuer, Luisa Cervantes-Barragan, Roland Züst, Burkhard Ludewig

**Affiliations:** 1 Institute of Immunobiology, Kantonsspital St. Gallen, St. Gallen, Switzerland; 2 Department of Pathology, Washington University School of Medicine, St. Louis, Missouri, United States of America; 3 Singapore Immunology Network, Agency for Science, Technology and Research, Singapore, Singapore; Leiden University Medical Center, Netherlands

## Abstract

Efficacy of antitumor vaccination depends to a large extent on antigen targeting to dendritic cells (DCs). Here, we assessed antitumor immunity induced by attenuated coronavirus vectors which exclusively target DCs in vivo and express either lymphocyte- or DC-activating cytokines in combination with a GFP-tagged model antigen. Tracking of *in vivo* transduced DCs revealed that vectors encoding for Fms-like tyrosine kinase 3 ligand (Flt3L) exhibited a higher capacity to induce DC maturation compared to vectors delivering IL-2 or IL-15. Moreover, Flt3L vectors more efficiently induced tumor-specific CD8^+^ T cells, expanded the epitope repertoire, and provided both prophylactic and therapeutic tumor immunity. In contrast, IL-2- or IL-15-encoding vectors showed a substantially lower efficacy in CD8^+^ T cell priming and failed to protect the host once tumors had been established. Thus, specific *in vivo* targeting of DCs with coronavirus vectors in conjunction with appropriate conditioning of the microenvironment through Flt3L represents an efficient strategy for the generation of therapeutic antitumor immunity.

## Introduction

Immunotherapy of cancer has moved from preclinical development into clinical practice [1,2]. For example, prophylactic vaccination using ‘non-self’ antigens such as virus-derived proteins from human papilloma viruses reduce the incidence of virus-induced tumors [3,4]. Moreover, the description of tumor-associated ‘self’ antigens [5,6] has opened new avenues for vaccination approaches that target eradication of established tumors cells. Indeed, recent phase III trials have shown that patient-specific cellular vaccines containing tumor antigens can improve survival of patients even with advanced disease [7,8]. However, since the production of tailored vaccines for individual patients requires laborious and expensive routines, generation of simple and efficient off-the-shelf reagents should be fostered.

Biological factors that make the development of therapeutic antitumor vaccines cumbersome include the immunosuppressive microenvironment within the tumor tissue itself [9,10] and remote inhibitory effects such as the preferential differentiation of T regulatory (Treg) cells [11,12]. It has been proposed that a combination of tumor antigens with immune-modulatory cytokines can overcome tumor-induced immunosuppression and/or –deviation [13]. Cytokines that foster activation of lymphocytes such as IL-2 or IL-15 have been evaluated in preclinical models and are currently tested in clinical studies [14–16] to augment tumor-specific immunity. Likewise, cytokines that act mainly on myeloid cells such as granulocyte macrophage colony-stimulating factor (GM-CSF) or Fms-like tyrosine kinase 3 ligand (Flt3L) have been shown to improve the efficacy of cancer vaccines [17,18]. However, cytokines generally exhibit a wide range of functions. For example, IL-2 is a potent stimulus for the activation of naïve T cells, but fosters at the same time activation-induced cell death of CD8^+^ effector T cells [19] and induces Treg cells in tumor patients [20]. Likewise, GM-CSF can foster generation and survival of myeloid suppressor cells [21,22]. Hence, it is important that cancer vaccines deliver such pleiotropic cytokines to those cells that optimally induce and maintain anticancer immune responses.

Dendritic cells (DCs) sample antigen in peripheral organs, and transport the immunogenic material to secondary lymphoid organs to initiate and maintain T and B cell responses [23]. DCs have to be appropriately stimulated to achieve full differentiation of T cells [24] and to overcome potential tolerizing stimuli within the microenvironment of secondary lymphoid organs [25]. Notably, it is important the DCs are directly activated through pattern-recognition receptors to achieve full maturation [26] and to successfully induce rejection of tumors [27]. 

Attenuated viral vectors exhibit several important advantages that make them attractive vaccine vehicles for antitumor vaccination. First, viral vaccines can be produced in large quantities and stored as off-the-shelf reagents. Second, viruses generally infect professional antigen presenting cells such as DCs, and third, viral infection triggers DC maturation [28]. We have recently suggested to utilize attenuated coronaviruses as vaccine vectors because (i) these positive-stranded RNA viruses replicate exclusively in the cytoplasm without a DNA intermediary, (ii) recent technological advances permit heavy attenuation without loss of immunogenicity, (iii) their large RNA genome offer a large cloning capacity, and (iv) both human and murine coronaviruses efficiently target DCs [29]. In a previous study, we found that murine coronavirus-based vectors can deliver multiple antigens and cytokines almost exclusively to CD11c^+^ DCs within secondary lymphoid organs [18]. Moreover, induction of CD8^+^ T cells directed against human tumor antigens and efficient transduction of human DCs with tumor antigen-recombinant human coronavirus 229E [18] indicate that coronavirus-mediated gene transfer to DCs should be considered as a versatile approach for antitumor vaccination. 

In the present study, we evaluated the impact of Flt3L or lymphoid cytokines co-expressed with a GFP-tagged model antigen in murine coronavirus vectors on antitumor immunity. We found that DCs transduced *in vivo* with vectors encoding for Flt3L efficiently activated tumor-specific CD8^+^ T cells, broadened the epitope repertoire, and secured therapeutic tumor immunity. Interestingly, IL-2 and IL-15 showed a significantly lower adjuvant effect on CD8^+^ T cell priming and failed to protect against established tumors indicating that coronavirus-mediated in vivo targeting of DCs in conjunction with the myeloid cell-stimulating cytokine Flt3L is well-suited to generate therapeutic antitumor immunity.

## Results

### Design of cytokine-expressing coronavirus vectors

**Figure 1:**
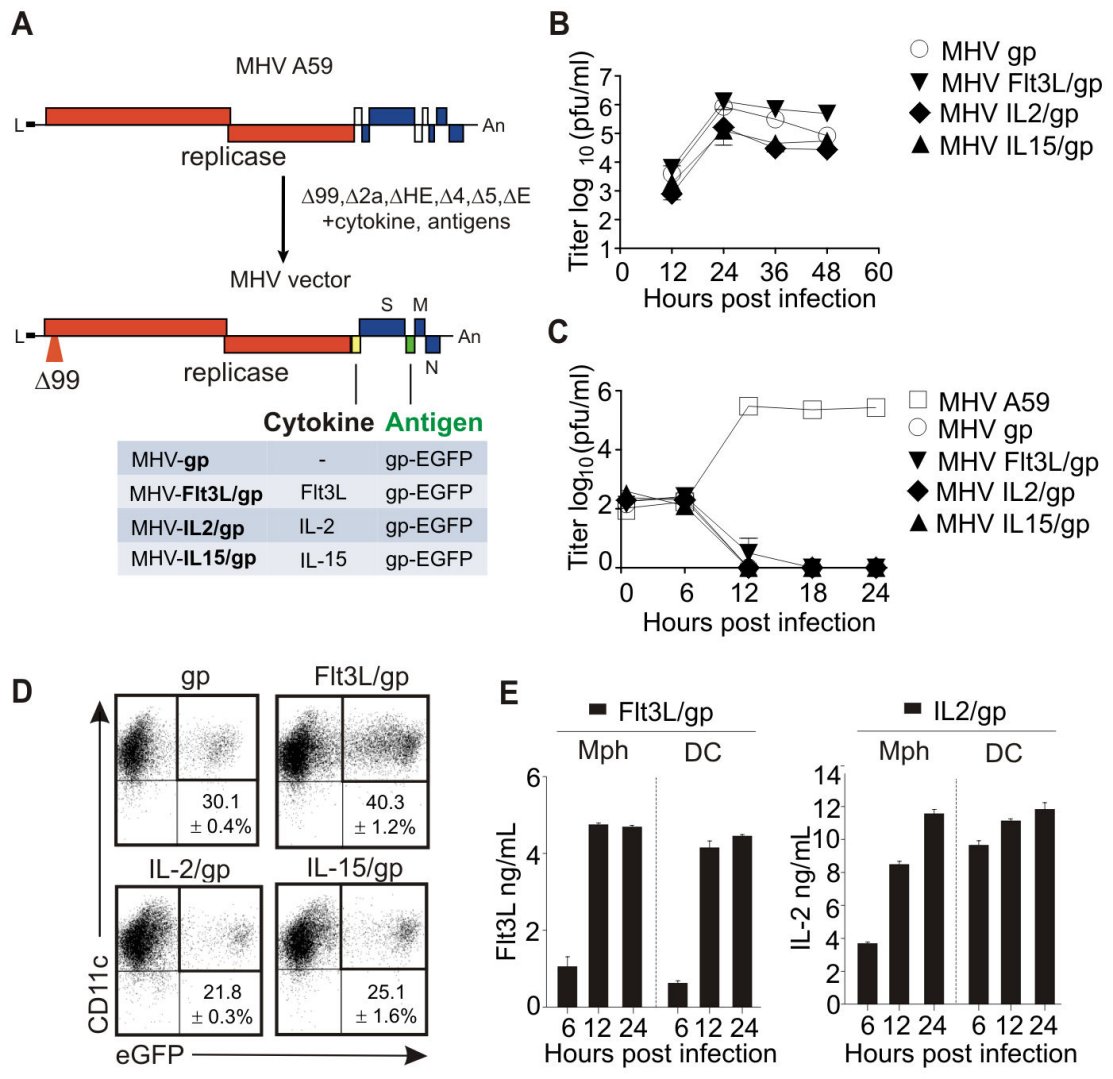
Generation and in vitro characterization of cytokine-encoding murine coronavirus vectors. (A) Schematic representation of MHV A59 genome and construction of cytokine-encoding vectors. (B, C) Growth kinetics of the indicated MHV vectors in 17ECl20 packaging cells (B) and macrophages (Mph) (C). Cells were infected at an MOI of 1 and titres in supernatants were determined at the indicated time points. (D) Transduction in bone marrow-derived DCs with murine coronavirus vectors. DCs from B6 mice were transduced with the indicated vectors at a multiplicity of infection (MOI) of 1. Cells were harvested 24 h later and EGFP expression on CD11c^+^ cells was assessed. Pooled data from three independent experiments with values indicating mean percentage ±SEM of EGFP^+^CD11c^+^ cells. (E) Cytokine production induced by Flt3L and IL-2 encoding vectors. DCs or macrophages were transduced at a MOI of 1 and concentration of cytokines in the supernatants was determined by ELISA. Representative data from one out of three independent experiments.

Coronavirus-based multigene vaccine vectors were designed on the basis of the mouse hepatitis virus (MHV) genome (Figure 1A). To achieve propagation deficiency and to maintain at the same time replication competence, MHV-encoded accessory genes (NS2, HE, gene4, gene5a) were deleted and the non-structural protein 1 (nsp1) was truncated by introducing a mutation that reduces MHV pathogenicity, but retains immunogenicity [30]. Moreover, the structural gene E was deleted to further hinder virion formation [31]. As surrogate tumor antigen, we used a fusion protein of the enhanced green fluorescent protein (EGFP) and the gp33 CD8^+^ T cell epitope derived from the lymphocytic choriomeningitis virus (LCMV) glycoprotein. To compare the adjuvant effects of cytokines acting mainly on myeloid cells versus lymphocytes, the genes encoding for murine Flt3L, IL-2 or IL-15 were inserted between the replicase and spike genes (Figure 1A). Propagation of MHV-based vectors was achieved in 17Cl1 packaging cells that provide the E protein in *trans* (Figure 1B). Importantly, the vectors failed to propagate in primary macrophages (Figure 1C) and DCs (not shown) in vitro, but efficiently delivered their antigen to DCs resulting in transduction rates of 20 - 40% (Figure 1D). *In vitro* transduced peritoneal macrophages and DCs rapidly produced Flt3L or IL-2 following transduction with the respective vector (Figure 1E), whereas IL-15 could not be detected following transduction with MHV-IL15/gp (not shown). Notably, IL-15 is known as a cytokine that is trans-presented by IL-15Rα [32] and hence can usually not be measured using conventional detection systems. Furthermore, we found that neither transduction with MHV-gp vector induced any of the three cytokines nor did the cytokine-expressing vectors elicit non-specific cytokine production (not shown). Thus, coronavirus vectors can specifically deliver antigen and different immune-modulatory cytokines to their major target cells.

### Coronavirus vector-induced dendritic cell maturation

**Figure 2:**
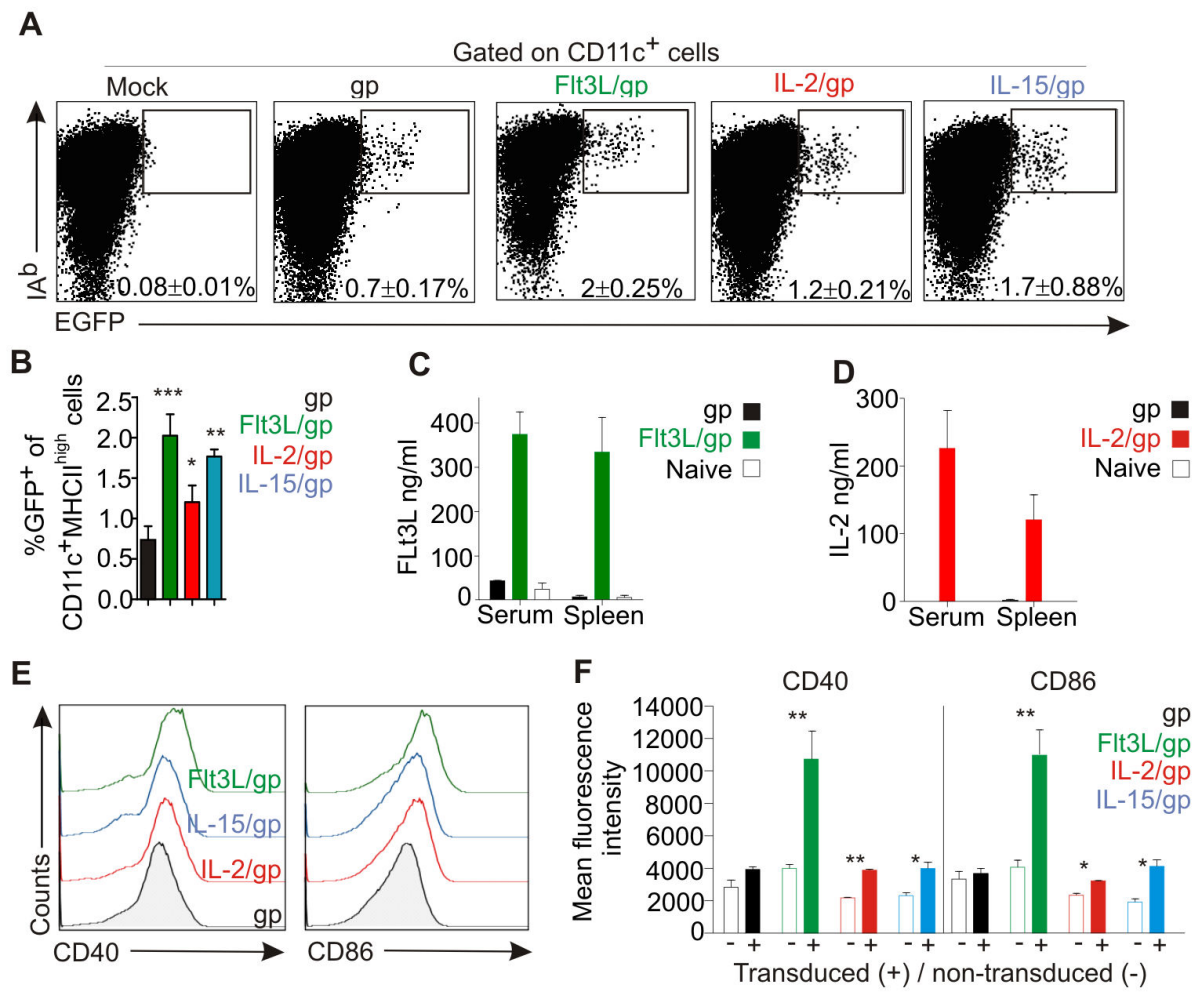
In vivo maturation of DCs following vaccination with coronavirus vectors. B6 mice were i.v. immunized with 10^6^ pfu of the indicated viral vectors or left untreated (mock). (A, B) Transduction of DCs as assessed by EGFP expression. Spleens were collected after 24 h, digested with collagenase and low-density cells were analyzed by flow cytometry. Values in dot plots (A) and bar graph (B) show the mean percentage ± SEM of EGFP^+^IA^bhigh^ cells gated on CD11c^+^ cells. Data from two independent experiments with three mice per group (n=6). (C, D) Cytokine concentration in serum and spleen homogenates at 24 h post immunization with Flt3L (C) and IL-2 (D) vectors. Pooled data from three independent experiments with three mice per group (mean ±SEM, n=9). (E) Representative histograms showing expression of the DC maturation markers CD40 and CD86 on EGFP^+^CD11c^+^ cells transduced with the indicated vectors. (F) Mean fluorescence values ±SEM (n=9) of CD40 and CD86 expression in transduced EGFP^+^CD11c^+^ cells (+) and non-transduced EGFP^+^CD11c^-^ cells (-) cells (*, p< 0.05; **, p< 0.01).

We have shown previously that the murine coronavirus preferentially infects macrophages and DCs in vivo [33]. Moreover, severe attenuation of MHV further focusses its target cell range to CD11c^+^ DCs [18]. This pronounced DC-specificity was not altered by insertion of Flt3L or lymphoid cytokines. Following i.v. application of 10^6^ vector particles into C57BL/6 (B6) mice, EGFP expression was detectable mainly in MHCII (IA^b^)^high^CD11c^+^ DCs (Figure 2A) whereby the cytokines roughly doubled *in vivo* transduction efficacy (Figure 2B). *In vivo* delivery of IL-2 (Figure 2C) and Flt3L (Figure 2D) resulted in high cytokine production after 48 h, whereas IL-15 could neither be detected in serum nor in spleen (not shown). Importantly, comparable to the transient elevation of GM-CSF levels following coronavirus-mediated delivery [18], both IL-2 and Flt3L levels had normalized at day 4 post application (not shown). Since maturation of DCs is critical for induction of protective immunity [23], we determined expression of the maturation markers CD40 and CD86 on *in vivo* transduced, EGFP^+^ DCs. As shown in Figure 2E, only the Flt3L-encoding vectors led to upregulation of both maturation markers. Interestingly, enhanced expression of CD40 and CD86 was restricted to those DCs that expressed EGFP (Figure 2F) indicating that the presence of the viral vector within DCs and direct production of the myeloid cell-stimulating cytokine Flt3L led to optimal DC maturation. 

### Coronavirus vector-delivered cytokines enhance CD8^+^ T cell induction

**Figure 3:**
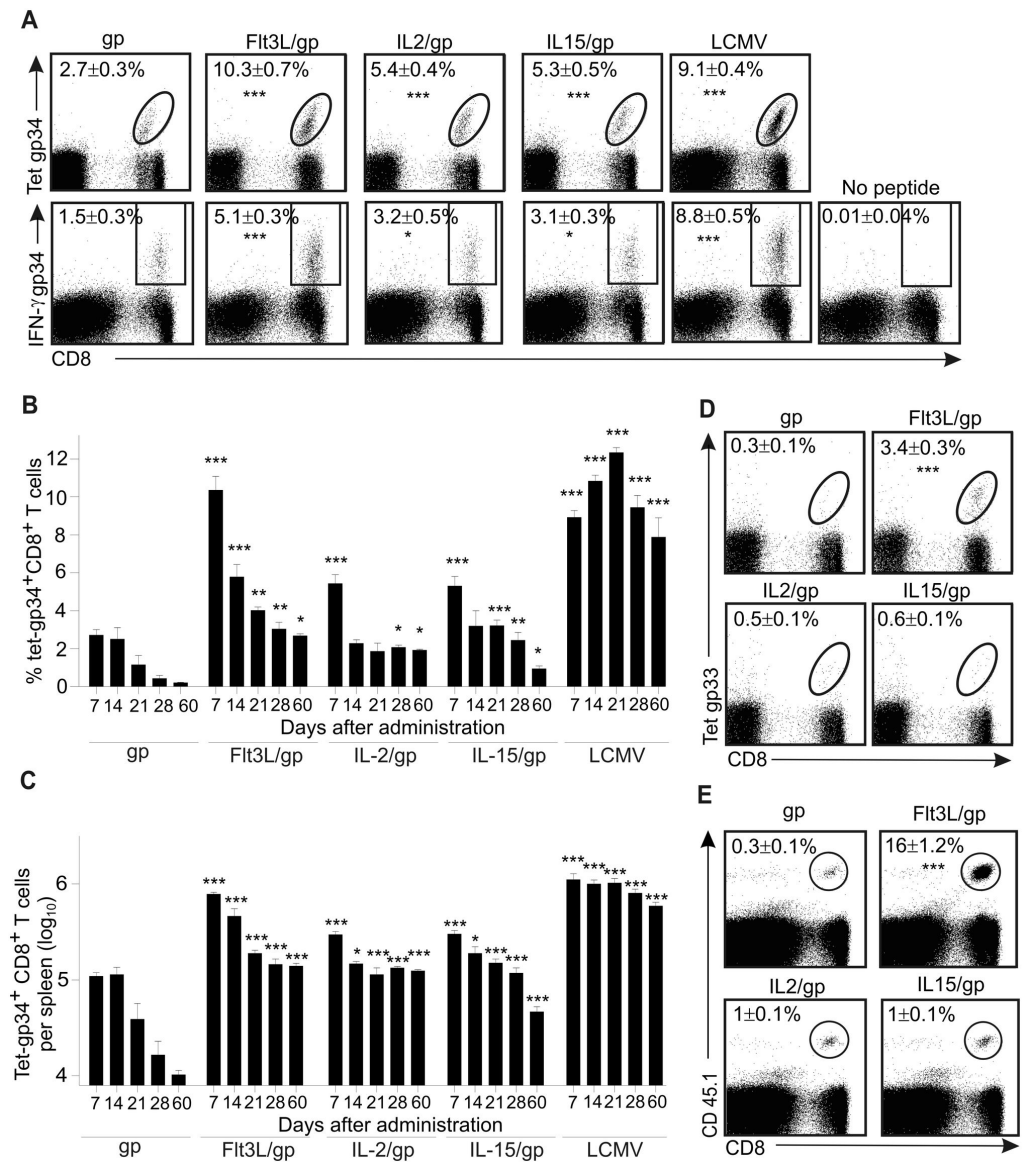
Evaluation of CD8^+^ T cell response induced by cytokine-encoding coronavirus vectors. (A) Induction of gp34-specific CD8^+^ T cells. B6 mice were immunized i.v. with 10^5^ pfu of the indicated vectors. At day 7 post immunization, splenocytes were analyzed for expression of CD8 and reactivity with H2-K^b^/gp34-tetramers, and were tested for gp34-specific IFN-γ production. Values indicate mean percentages of tet^+^ cells ± SEM (upper row) or mean percentages of IFN-γ^+^ cells ± SEM (lower row) in the CD8^+^ T-cell compartment (pooled data of 3 independent experiments, n=12 mice). (B and C) Duration of vector-induced CD8^+^ T cell responses. B6 mice were immunized i.v. with 10^5^ pfu of the indicated vectors or infected with 200 pfu of LCMV WE. Frequencies (B) and total numbers (C) of splenic CD8^+^ tet-gp34-binding T cells were determined at the indicated time points (mean percentages or total numbers of tet-gp34^+^ cells ±SEM, n=6-12 mice per time point); nd, not detectable. (D) Induction of gp33-specific CD8^+^ T cells as determined by tetramer analysis on day 7 post i.v. immunization with the indicated vectors (mean percentages of tet-gp33^+^ cells ± SEM, n=8 mice). (E) Expansion of gp33-specific P14 TCR transgenic CD8^+^ T cells. One day before immunization with 10^5^ pfu of the indicated vectors, CD45.2^+^ B6 mice had received 10^5^ CD45.1^+^ P14 splenocytes. Expansion of CD45.1^+^CD8^+^ T cells in spleens was assessed on day 7 post immunization (mean percentages of CD45.1^+^CD8^+^ T cells ±SEM, n=8 mice) (*, p< 0.05; **, p< 0.01; ***, p< 0.001; comparison with gp control vector).

To compare the adjuvant effects of the different cytokines encoded by the coronavirus vectors, we first assessed magnitude and duration of transgene-specific CD8^+^ T cell activation. In addition, to determine cytokine-mediated changes in epitope usage, we monitored CD8^+^ T cell responses against the H2-D^b^-binding gp33-41 [34] and the H2-K^b^-binding gp34-41 [35] epitopes which are both present in the gp33-EGFP transgene. Using i.v. application of 10^5^ pfu of each vector, we found that induction of gp34-specific CD8^+^ T cells responses was best supported by Flt3L leading to superior expansion of tetramer-binding CD8^+^ T cells and differentiation towards IFN-γ-producing effector T cells (Figure 3A). Importantly, initial expansion of gp34-specific CD8^+^ T cells following vaccination with MHV-GP/Flt3 was comparable to the responses induced by the fully replication-competent LCMV (Figure 3B and 3C). Surprisingly, although IL-2 and IL-15 have been described as cytokines that support generation of memory T cells [36], the Flt3L-encoding vector better supported the persistence of transgene-specific CD8^+^ T cells (Figure 3B and 3C). As shown previously [18], MHV-gp vectors failed to raise a substantial response against the H2-D^b^-binding gp33 epitope (Figure 3D). Likewise, the vectors encoding for IL-2 or IL-15 did not elicit a pronounced CD8^+^ T cell response against the gp33 epitope, whereas vector-encoded Flt3L promoted a broadening of the antigen reactivity towards gp33 (Figure 3D). This finding was corroborated using adoptive transfer of gp33-specific P14 TCR transgenic T cells one day before application of the vectors. Again, Flt3L very efficiently supported the expansion of gp33-specific CD8^+^ T cells (Figure 3E) indicating that this cytokine functions as an optimal adjuvant for DC-specific targeting approaches.

### Tumor immunity elicited by coronavirus vectors

**Figure 4:**
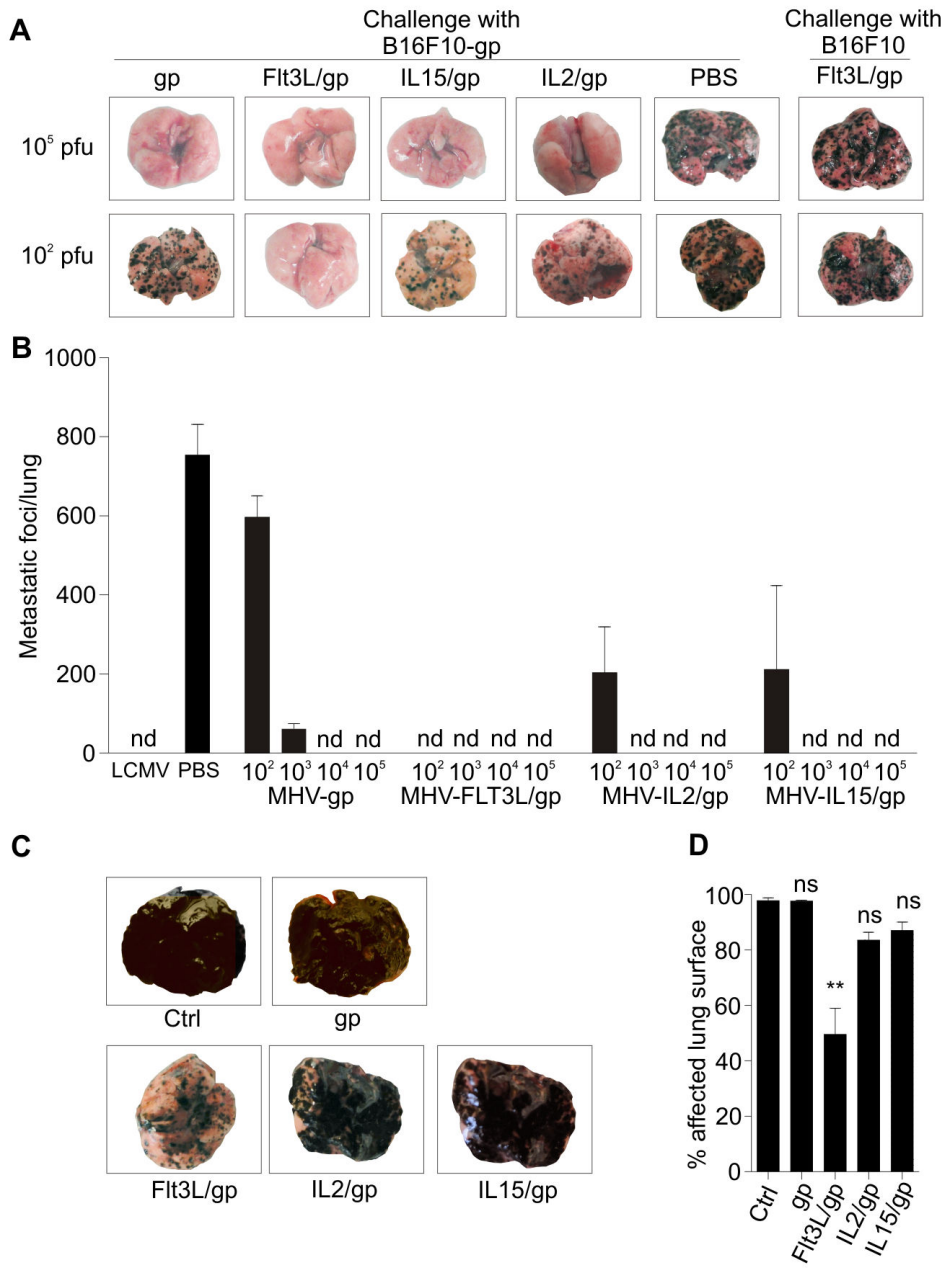
Coronavirus vector-induced immunity against a metastasizing tumor. (A, B) B6 mice were i.v. immunized with the indicated doses of the different vectors or infected with 200 pfu LCMV WE. PBS was administered as negative control. Seven days later mice were challenged with 5×10^5^ B16F10-gp melanoma cells or the parental B16F10 cells. (A) Representative microphotographs of lungs on day 12 post tumor inoculation. (B) Number of metastatic foci per lung was determined 12 days after challenge (pooled data from three independent experiments, mean ±SEM, n=6-9 mice per time point). (C, D) Assessment of therapeutic tumor immunity. B6 mice received 5×10^5^ B16F10-gp melanoma cells i.v. at day 0 and were vaccinated 10 days later with 10^5^ pfu of the indicated vectors or left untreated. (C) Representative microphotographs of affected lungs on day 20 after tumor inoculation. (D) Disease severity was quantified by black pixel counting on lung surfaces on day 20. Values represent mean percentage ±SEM (n=6-8 mice) of affected lung surface. Statistical analysis in (D) was performed using one way ANOVA with Tukey’s post analysis (**, p< 0.01; ns, non-significant).

Induction of potent CD8^+^ T cell responses against tumor antigens is critical to establish and maintain tumor immunity [37]. To assess the impact of lymphoid versus Flt3L-mediated adjuvant effects on protective tumor immunity, we utilized two different tumor models which provide compatibility with the LCMV-GP system due to the expression of a gp33 minigene [38,39]. In the first model, we assessed metastatic growth of murine B16F10 melanoma cells. To this end, 5×10^5^ B16F10-GP or parental B16F10 cells were applied to B6 mice which resulted in metastatic growth of tumor cells in lungs (Figure 4A). Vaccination of recipient mice on day 7 before tumor challenge with 10^5^ pfu of the different coronavirus vectors had completely blocked growth of B16F10-GP tumor cells, whereas formation of metastatic foci in lungs by parental B16F10 cells was not affected (Figure 4A). Moreover, prophylactic vaccination with 10^2^ pfu of IL-2 or IL-15 encoding vectors reduced the tumor burden, whereas the same dose of Flt3L vector completely prevented tumor growth (Figure 4A). Careful titration of the cytokine-encoding vector doses and compilation of several series of experiments in the prophylactic vaccination setting revealed that indeed only 10^2^ viral particles of MHV-gp/Flt3L were sufficient to completely protect the mice from tumor challenge (Figure 4B). To assess whether cytokine-encoding vectors can elicit therapeutic tumor immunity, mice were first inoculated with B16F10-GP tumors and vaccinated 10 days later with 10^5^ pfu of the different coronavirus vectors. Again ten days later, i.e. at day 20 post tumor inoculation, melanoma cells had almost completely covered the whole lung surface in control and MHV-gp vaccinated mice, whereas therapeutic vaccination with cytokine-encoding vectors had reduced the tumor load (Figure 4C). Importantly, determination of affected lung surface revealed that only vaccination with the Flt3L-encoding vector was able to significantly reduce the tumor load (Figure 4D).

**Figure 5:**
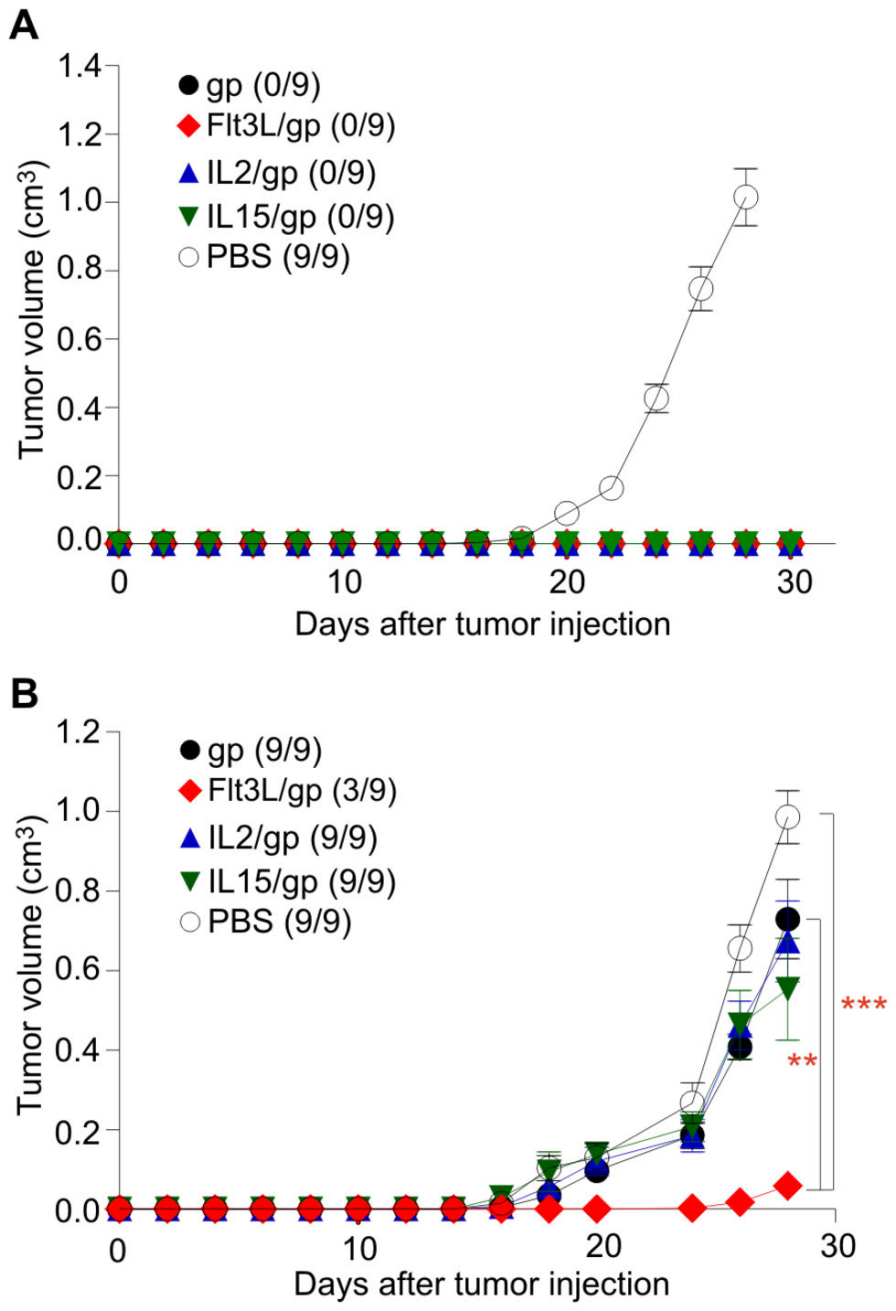
Prophylactic and therapeutic tumor immunity against a peripheral solid tumor. (A) B6 mice were i.v. immunized with 10^5^ pfu of the different vectors or received PBS as control. Seven days later, 5×10^5^ gp-recombinant Lewis lung carcinoma cells were injected s.c. on the left flank. (B) Assessment of therapeutic tumor immunity induced by 10^5^ pfu of the different vectors applied on day 4 post s.c. inoculation with 5×10^5^ gp-recombinant Lewis lung carcinoma cells. Tumor growth was monitored on the indicated days. Values indicate mean tumor volume ±SEM (n=9 mice). Values in parentheses indicate number of growing tumors of inoculated tumors. Statistical analysis in (B) was performed using one way ANOVA with Bonferroni multiple comparison test (**, p< 0.01; ***, p< 0.001; ns, non-significant).

Clearance of rapidly growing metastatic tumors is certainly a challenge for the immune system. However, metastatic tumors such as the B16 melanoma cells can reach secondary lymphoid organs and thereby contribute – most likely due to their MHC class I expression – to the amplification of antitumor CD8^+^ T cells [40]. In contrast, tumors which arise in peripheral tissues can escape immune surveillance in the absence of appropriately activated T cells [41]. To assess whether coronavirus vector-based vaccination can prevent growth of such peripherally growing tumors, we applied 5×10^5^ LCMV-GP recombinant Lewis lung carcinoma (LLC) cells s.c. into prophylactically vaccinated B6 mice. As shown in Figure 5A, all coronavirus-based vectors were able to protect the animals under these conditions. However, when we assessed the efficacy of the vaccines in a therapeutic approach, i.e. applying the vaccines on day 4 post tumor inoculation, only FLt3L-encoding vectors achieved an almost complete block of tumor growth (Figure 5B). Taken together, these results indicate that different cytokines can improve immunogenicity of coronavirus vectors. However, maturation of DCs through vector-encoded cytokines that preferentially activate myeloid cells is essential for the generation of therapeutic tumor immunity.

## Discussion

Recent clinical trials have shown that viral vector-based vaccination against cancer is feasible, safe and suitable to achieve protective tumor immunity [42]. For example, a randomized, placebo-controlled study in patients suffering from castration-resistant prostate carcinoma (n=125) showed that a heterologous prime-boost regimen with different poxvirus vectors delivering prostate-specific antigen increased the median survival by 8.5 months [43]. Notably, this approach using an off-the-shelf vaccine yielded an efficacy comparable to the personalized cellular vaccine Sipuleucel which increases survival of patients suffering from incurable prostate cancer by 4.1 months [7]. Nonetheless, the high immunogenicity of viral vectors results in the induction of antiviral immunity which makes heterologous prime-boost schemes necessary to guarantee repeated exposure to the target antigen [44]. Hence, coronavirus vectors based on human DC-targeting common cold viruses [18], represent a potential asset for future clinical trials that utilize heterologous prime-boost approaches. Importantly, vaccination with attenuated murine coronavirus vectors permits usage of the vectors even in homologous prime-boost schemes hence leading to the maintenance of effector T cells at high frequencies [18]. Since human coronaviruses causing common cold frequently re-infect their hosts [45], it is possible that this human viral vector family may be utilized as well in homologous prime-boost approaches.

The ability of DCs to induce antigen-specific tumor immunity can be enhanced by immunological adjuvants such as toll-like receptor (TLR) ligands which lead to optimal immune activation only when DCs are stimulated directly through their TLRs [26,27]. Likewise, cytokines that stimulate the myeloid compartment such as the granulocyte-macrophage colony-stimulating factor (GM-CSF), facilitate DC maturation and foster their survival [46]. Our previous study revealed the importance of DC-specific GM-CSF delivery for both DC maturation and prolongation of antigen presentation through improved DC survival leading to sustained CD8^+^ T cells immunity against viral infection and tumors [18]. Here, we found that a second myeloid cell-stimulating cytokine very efficiently enhanced the immunogenicity of coronavirus vectors. Notably, the Flt3L vectors were even more effective than the GM-CSF vectors in preventing metastatic growth of melanoma cells [18]. Moreover, MHV-Flt3L/gp vectors, but not GM-CSF-encoding vectors [18], elicited a broadening of the epitope repertoire with presentation of two epitopes from the gp33 minigene. Thus, the iterative approach of coronavirus vector optimization using a single model antigen and standardized read-out methods facilitates the definition of cytokines that display optimal adjuvant effects for this vaccine.

In this study, we also evaluated the adjuvant effect of cytokines that act more downstream in the immune activation cascade. The lymphocyte-stimulating cytokines IL-2 and IL-15 are known to enhance proliferation of T cells, to foster differentiation of CD8^+^ T cells and to control formation of T cell memory [36]. However, despite sharing two out of three of their receptor units (common gamma chain and IL-2/IL-15β-R) these cytokines impact differently on T cell differentiation. IL-2 is most important for the early expansion of T cells and via activation-induced cell death, the limitation of the T cell overshoot [47]. IL-15, on the other hand, is essential for survival of high-affinity T cells during the memory phase [48] and ensures thereby the maintenance of protective T cell memory responses. It is important to note that both IL-2 and IL-15 are highly efficient adjuvants that have been shown to enhance tumor-specific T cell responses [36]. Our data confirm that IL-2 and IL-15 improve CD8^+^ T cell activation and induction of prophylactic tumor immunity, also when incorporated in a coronavirus-based vaccine that delivers the cytokines directly to DCs *in vivo*. 

It is interesting to note that neither the secreted and systemically acting IL-2 nor the more locally acting, trans-presented IL-15 reached the efficacy of the DC-stimulating Flt3L. Flt3L is a highly immunostimulatory cytokine that supports, for example, control of parasitic infections both in mice and humans [49]. Moreover, Flt3L efficiently mobilizes DC precursors, for example in humans with metastatic colon cancer [50]. The results of the present study suggest that high vaccine immunogenicity can be achieved through direct transduction of DCs via a coronavirus vector with concomitant delivery of a potent DC maturation factor such as Flt3L. Clearly, further analyses are warranted to reveal the molecular events involved in Flt3L-mediated adjuvant effects in this system. In addition, it is possible that a combination of different cytokines delivered by coronavirus vectors in homologous prime-boost regimen, e.g. Flt3L followed by IL-15 vectors or *vice versa*, will result in a further improvement of coronavirus vector-induced antitumor immunity.

In conclusion, incorporation of cytokines into coronavirus vectors substantially improves their performance. Since these vectors almost exclusively target DCs, expression of cytokines that facilitate DC maturation and foster their survival such as GM-CSF or Flt3L, is highly efficient and facilitates generation and maintenance of antitumor CD8^+^ T cells. Therefore, coronavirus-based vectors that express DC-stimulating cytokines should be further developed for their utilization in therapeutic cancer vaccination. 

## Materials and Methods

### Ethics statement

Experiments were performed in accordance with federal and cantonal guidelines under permission numbers SG09/92, SG11/06, and SG11/10 following review and approval by the Cantonal Veterinary Office (St. Gallen, Switzerland). 

### Mice, cells and viruses

C57BL/6 mice were obtained from Charles River Laboratories (Sulzfeld Germany). P14 TCR transgenic mice were obtained from the Swiss Immunological Mutant Mouse Repository (Zurich, Switzerland). All mice were maintained in individual and ventilated cages and were used between 6 and 9 weeks of age. 17Cl1 cells were a kind gift from S. G. Sawicki (Medical University of Ohio, Toledo, OH). The LCMV WE strain was obtained from R. M. Zinkernagel (Universität Zürich, Switzerland). Titration of MHV vectors has been performed as described previously [33]. 

### Cloning and generation of recombinant MHV-based vectors

Generation of recombinant MHV vectors is based on reverse genetic systems established for MHV-A59 [51]. Molecular cloning and production of recombinant coronavirus particles was performed as previously described [18]. 

### Isolation of dendritic cells and macrophages, flow cytometry

Bone marrow–derived DCs were generated by culturing erythrocyte-depleted bone marrow cells for 6 to 7 days in the presence of GM-CSF containing supernatant from the cell line X63-GM-CSF (kindly provided by Antonius Rolink, University of Basel, Basel, Switzerland) as described previously [33]. DCs were further purified using Optiprep density gradient centrifugation. Splenocytes were obtained from spleens of B6 mice following digestion with collagenase type II for 20 min at 37°C and resuspended in RPMI/5% FCS. For isolation of the low density cells, splenocytes were resuspended in PBS supplemented with 2% FCS, 2 mM EDTA and overlaid on 20% Optiprep density gradient medium (Sigma-Aldrich Co. Basel, Switzerland). After centrifugation at 700×g for 15 min, low density cells were recovered from the interface and resuspended in RPMI/5% FCS. Cells were stained with different lineage markers and analyzed for EGFP expression with a FACSCanto flow cytometer using the FACS Diva software (BD Biosciences). Antibodies used in this study were purchased from BD Biosciences Pharmingen (CD11c-PE), or Biolegend (CD40-APC, CD86-APC, IA^b^-Alexa fluor 647 ). 

### Tetramer analysis and intracellular cytokine staining

Enumeration of virus- specific CD8^+^ T cells and ex vivo production of IFN-γ were determined by tetramer staining and intracellular cytokine staining, respectively, as described previously [30]. Organs were removed at the indicated time points following immunization with MHV-based vectors. Tetramers were synthesized and applied for staining of blood and spleen samples as previously described [52]. For intracellular cytokine staining, single cell suspensions of 10^6^ splenocytes were incubated for 5 h at 37°C in 96-well round-bottom plates in 200 μl culture medium containing Brefeldin A (Sigma). Cells were stimulated with phorbolmyristateacetate (PMA, 50 ng/ml) and ionomycin (500 ng/ml) (both purchased from Sigma, Buchs, Switzerland) as positive control or left untreated as a negative control. For analysis of peptide-specific responses, cells were stimulated with 10^-6^ M of the indicated peptides. Cells were further surface-stained with CD8-APC (eBiosciences), permeabilized with Cytofix-Cytoperm (BD Biosciences) and intracellularly-stained with IFN-γ-PE. The percentage of tet^+^CD8^+^ T cells and CD8^+^ T cells producing IFN-γ was determined using a FACSCanto flow cytometer using the FACS Diva software (BD Biosciences). GP33 (KAVYNFATC) and GP34 (AVYNFATC) peptides were purchased from Neosystem (Strasbourg, France). 

### Tumor models

B16F10-GP melanoma cells expressing the LCMV gp33 epitope [38] and parental B16F10 cells were kindly provided by Dr. H. Pircher (University of Freiburg, Germany). The B16F10-GP melanoma cells were cultivated under G418 (200 µg/ml) (Life Technologies, Gaithersburg, MD) selection. For prophylactic vaccination experiments, mice were immunized with MHV-based vectors seven days before i.v. tumor challenge with 5×10^5^ tumor cells. Protection was determined as numbers of metastasic foci per lung on day twelve post tumor inoculation. For therapeutic vaccination, mice received 5×10^5^ tumor cells i.v. and were immunized ten days later with the indicated vectors. Tumor clearance was determined on day twenty and recorded as percentage of affected lung surface. 

LLC cells expressing H-2D^b^-restricted peptide 33-41 of the LCMV glycoprotein [39] were kindly provided by Dr. Franca Ronchese (University of Wellington, New Zealand). Efficacy of prophylactic vaccination was assessed by immunizing B6 mice i.v. with 10^5^ pfu coronavirus vectors seven days before s.c. challenge with 5×10^5^ tumor cells in the left flank. Therapeutic vaccination was done using i.v. application of 10^5^ pfu coronavirus vectors in B6 mice which had received 5×10^5^ tumor s.c. in the left flank four days previously. At the indicated time points, tumor volume was recorded as V=π x abc/6, whereby a, b and c are the orthogonal diameters. 

### Statistical Analysis

Statistical analyses were performed with Graphpad Prism 5.0 using non-paired, two tailed Student’s t test. Comparison between different groups was done using one way ANOVA with Tukey’s post test or with Bonferroni multiple comparison test as indicated. Statistical significance was defined as *p* < 0.05. 
